# Can social media encourage diabetes self-screenings? A randomized controlled trial with Indonesian Facebook users

**DOI:** 10.1038/s41746-024-01246-x

**Published:** 2024-09-13

**Authors:** Manuela Fritz, Michael Grimm, Ingmar Weber, Elad Yom-Tov, Benedictus Praditya

**Affiliations:** 1 University of Passau, Department of Economics, Passau, Germany; 2 Technical University Munich, School of Social Science and Technology, Munich, Germany; 3 IZA, Bonn, Germany; 4 RWI Research Network, Essen, Germany; 5 Saarland University, Department of Computer Science, Saarbruecken, Germany; 6 Bar Ilan University, Department of Computer Science, Ramat Gan, Israel; 7 Xiaomi Indonesia, DKI Jakarta, Indonesia

**Keywords:** Population screening, Risk factors, Health care economics

## Abstract

Nudging individuals without obvious symptoms of non-communicable diseases (NCDs) to undergo a health screening remains a challenge, especially in middle-income countries, where NCD awareness is low but the incidence is high. We assess whether an awareness campaign implemented on Facebook can encourage individuals in Indonesia to undergo an online diabetes self-screening. We use Facebook’s advertisement function to randomly distribute graphical ads related to the risk and consequences of diabetes. Depending on their risk score, participants receive a recommendation to undergo a professional screening. We were able to reach almost 300,000 individuals in only three weeks. More than 1400 individuals completed the screening, inducing costs of about US*$*0.75 per person. The two ads labeled “diabetes consequences” and “shock” outperform all other ads. A follow-up survey shows that many high-risk respondents have scheduled a professional screening. A cost-effectiveness analysis suggests that our campaign can diagnose an additional person with diabetes for about US*$*9.

## Introduction

Non-communicable diseases (NCDs), such as cardiovascular diseases, diabetes, and cancer, have overtaken infectious diseases as the leading cause of death worldwide^1^. Screening for metabolic NCD risk factors, such as high blood sugar and blood pressure, provides an effective tool to prevent more severe long-term health consequences. Also, behavioral risk factors, such as smoking, drinking, unhealthy diets, and a lack of physical activity, can be addressed once an individual is aware of its personal risk. Yet, nudging individuals to undergo such a screening in case of no apparent symptoms remains a challenge. This holds especially true in low- and middle-income countries (LMICs), where health literacy and the awareness of and screening for NCDs remain limited^2–4^. At the same time, NCDs are increasing at an unprecedented rate in many LMICs, requiring innovative solutions to increase NCD screening^5–7^.

To increase NCD awareness and screening in LMICs, the World Health Organization (WHO) promotes mass media awareness campaigns as a cost-effective instrument^8,9^. Yet, their focus is largely on traditional media such as TV, radio and print, whereas public health campaigns via social media advertising remain unmentioned. Social media public health campaigns and health advertisements have been shown to be promising to address a variety of health aspects and health behaviors. For example, social media public health campaigns have been used to address vaccination rates^10–14^, Covid-19 infections^15^, drinking during pregnancy^16^, smoking cessation^17^, sexual behaviors^18^, food choices and physical activity^19,20^.

Our study adds to this literature, but goes beyond these studies in multiple aspects. First, the major share of these campaigns is implemented and evaluated in high-income countries and addresses health topics of which the general public is broadly aware off. The question of whether such social media health campaigns work similarly well in LMIC contexts, especially if they address a disease for which there is little knowledge and awareness^4,21,22^, remains unanswered and we address this research gap. Thereby, we also directly speak to the literature that evaluates which other means and nudges (e.g., messages through community leaders or reminders) are effective in LMICs in encouraging better health-related outcomes and behavior^23,24^.

Second, most campaigns are limited to the pure provision of information and do not observe and engage viewers in concrete measurable actions other than those happening online (e.g., clicks or likes). Instead, users in our campaign were redirected to our campaign website, on which they could engage in an actual screening activity. Moreover, through a follow-up survey with part of the participants, we elicited behavior that happened (offline) after the campaign exposure. Notable exceptions to mention here are a study on Covid-19 infections^15^, which also expands the research design to offline measurements of user mobility and actual infection rates, and a study on HPV vaccination^14^ which measures actual vaccination rates.

Lastly, only a limited number of studies address the aspect of cost-effectiveness, despite the major advantage of online campaigns being cheap in comparison to other mass media campaigns. More specifically, while some studies evaluate the cost per person reached or the cost per person recruited with such campaigns^25,26^, they do not go as far as evaluating the cost per actual diagnosed case or prevented case. Hence, we conduct a cost-effectiveness analysis of our campaign to provide insights about the cost-saving potential of social media public health campaigns (beyond the cost per person reached), which is especially relevant in contexts of limited public health budgets as it is the case in Indonesia and in many other LMICs^27^.

We design, implement, and evaluate a diabetes health campaign and assess whether health advertisements (“ads”) distributed via Facebook can serve as a promising instrument to foster the individual decision to undergo a diabetes risk screening in Indonesia. Indonesia is a relevant setting for our campaign since diabetes is currently the third leading cause of death^28^. Moreover, the country ranks fifth in the list of absolute numbers of diabetes cases and third among the countries with the highest number of undiagnosed cases worldwide. In 2021, more than 19 million individuals were estimated to be living with the disease in Indonesia, with more than 70% of the cases remaining undiagnosed^29^. At the same time, the usage rate of Facebook is high, which lends itself as a perfect showcase to study whether social media campaigns are suitable to encourage people to engage in preventive health behavior such as diabetes screening. Given this setting, our results are relevant for many other middle-income countries with similar high rates of diabetes and large numbers of Facebook users, such as other countries in Southeast Asia, as well as for example India, Brazil, Mexico or Pakistan.

Facebook is becoming an increasingly relevant tool for scientific research, especially in terms of implementing randomized controlled trials (RCTs) with a large outreach^12,13,15,30^. Given the platform’s possibilities to specify concrete population targeting criteria and using Facebook’s A/B split test function, it allows us to target our campaign to Facebook users in the cities with the highest diabetes rates in Indonesia (Jakarta and Yogyakarta), and to provide causal evidence on the effectiveness of different ad designs. Specifically, we use an RCT on Facebook and distribute ads that differ in their framing, i.e., in their message and graphical design, but equally invite viewers to visit our campaign website and to complete a diabetes self-screening. We are especially interested in whether loss-framed, i.e., shocking, messages work better than more neutral ads. Theoretical work by Rothman et al.^31,32^ suggests that loss-framed or shocking messages should be more effective in inducing health behaviors that might be perceived as risky (i.e., have an uncertain outcome), such as disease detection activities. Following this argument, we hypothesize that a diabetes awareness campaign that encourages diabetes screening might be most effective if a shocking or loss-framed perspective is taken and investigate this proposition experimentally. Thereby, we also add to the empirical literature that explores what kind of information, framings or pictorial content drive health-related decisions^33–39^, in particular health screening activities^40–42^. Specifically, we provide evidence about which ads can effectively nudge individuals to learn about their risk of having or developing diabetes in a country where general disease awareness is low.

We then assess whether the most persuasive ad is good enough to design a cost-effective awareness campaign. Hence, in this second part of our analysis, we are interested in whether a campaign based on the cost and effectiveness parameters of the best-performing ad can be considered a cost-effective public health intervention. To this end, we follow-up with a subset of participants that completed the self-screening and investigate their compliance rate with the recommendation to schedule an appointment for a professional screening if they were found to be at high risk.

## Results

### Campaign outreach and engagement

From March 15 until April 5, 2022, we ran a diabetes health campaign entitled “Ada Gula, Ada Diabetes”. The title is related to the traditional Indonesian saying “Ada gula, ada semut”, which literally means “When there is sugar, there must be ants”. Figuratively, the saying means that for every action there is an equal and opposite reaction. Our adapted campaign name hence figuratively interprets diabetes as the reaction to too much sugar – also in relation to the fact that diabetes is known as “Sakit Gula” (“sugar disease” or “sugar sickness”) in Indonesia. We ran the campaign in Jakarta and Yogyakarta and used five different ads, two of which took on a loss-framed and rather disquieting perspective, with the remaining three referring to the family, religion, and the local diabetes prevalence rate (see Methods for a detailed description of the campaign and ads). After clicking on one of the ads, users were re-directed to our campaign website, where they were offered the opportunity to complete a diabetes risk screening questionnaire similar to the diabetes risk test of the American Diabetes Association and the diabetes FINDRISC (Finnish Diabetes Risk Score) screening test but adapted to the Indonesian population (see Methods section for details and Supplementary Tables 1 and 2 for the complete questionnaire). Based on the individual answers, a risk score between 0 and 16 points was calculated and participants received an assessment of their personal risk. Additionally, the assessment contained recommendations on how to keep the risk low, how the diabetes risk can be reduced and to visit a health center or a physician if the risk score was too high. Six weeks after the end of the campaign we sent a follow-up survey to (voluntarily left) e-mail addresses to elicit information about actual compliance with the recommendations received.

Table 1 presents the Facebook engagement statistics of our campaign by age, gender, and location (statistics by ad are presented in Supplementary Table 3). These descriptive statistics show that our Facebook campaign can be deemed effective in distributing diabetes-related ads and reaching the general public: Within only three weeks, we reached in total 286,776 individuals with our campaign, generated 758,977 impressions (distinct views of the ads) and 5274 link clicks. This amounts to a click rate of 1.84% (relative to the number of reached individuals), which is higher than the rates achieved in studies with a similar setup, for example in Tjaden et al.^12^ (1.7%), Choi et al.^43^ (1.4%) or Orazi^30^ (0.2%). Overall, we spent approximately US*$*1060 and the campaign resulted in 2052 started and 1469 completed screening questionnaires, implying a conversion-to-reach rate of 0.51% (1469/286,776) and a conversion-to-click rate of 27.85% (1469/5274). Moreover, this relates to a cost of around US*$*0.75 per person conducting such a self-screening. The age and gender patterns reflect the Indonesian Facebook user rates, with slightly more men than women using the platform and the elderly having the lowest user rates^44,45^.

**Table 1:** Outreach of the Facebook campaign

	(1)	(2)	(3)	(4)	(5)
	Reach	Impressions	Link clicks	Expenditure	Conversions
	(# of persons)	(# of distinct views)	(# of ad clicks)	(in US*$*)	(compl. quest.)
**Total**	**286,776**	**758,977**	**5274**	**1066.04**	**1469**
*By Gender*					
Male	160,560 (56%)	433,677 (57%)	2725 (52%)	570.91 (54%)	754 (51%)
Female	126,216 (44%)	325,300 (43%)	2549 (48%)	495.13 (46%)	715 (49%)
*By Age*					
Below 45	136,500 (48%)	342,729 (45%)	1646 (31%)	372.01 (35%)	466 (32%)
45–54	98,060 (34%)	271,914 (36%)	2091 (40%)	421.23 (40%)	686 (47%)
55–64	32,820 (11%)	90,314 (12%)	978 (19%)	188.42 (18%)	238 (16%)
65+	19,396 (7%)	54,020 (7%)	559 (11%)	83.37 (8%)	79 (5%)
*By Location*^1^					
Jakarta	145,528 (51%)	321,154 (42%)	2834 (54%)	526.75 (49%)	876 (60%)
Yogyakarta	141,248 (49%)	437,823 (58%)	2440 (46%)	539.29 (51%)	567 (40%)

^1^The number of completed screening questionnaires by location does add up to 1443 and not to 1469 since for 26 completed questionnaires tracking was restricted and the referring ad and thus the location could not be determined.

Due to changes in Apple’s data policy, Facebook is unable to track users who opted out of tracking under iOS 14 or users who prohibit tracking in any other form and therefore relies on statistical modeling to estimate the total number of conversions^46^. Moreover, Facebook is unable to differentiate by age or gender once the users leave the platform and thus only provides aggregated data on conversions. Hence, for the results in terms of conversions (Column (5)), we rely on the more accurate data that was collected directly on our campaign website from which we could extract – without any loss or modeling – the absolute number of completed (and started) screening questionnaires by age, gender, and location.

### Screening participation

Once redirected to our campaign website, participants could fill out the screening questionnaire. We used Facebook’s dynamic URL parameters^47^ to generate ad-specific referrer links containing information about the ad id, ad name, and ad placement. These URL parameters could then be read out whenever an individual started to fill out the screening questionnaire. For those individuals using an Apple device who opted out of tracking, the ad-specific URL parameters within the referrer link would not be displayed. However, given that a vast majority of smartphone users in Indonesia rely on an Android system, only 26 (out of 1469) completed screening questionnaires could not be linked to the ad from which users were redirected to our campaign website.

Respondents had the possibility to complete the screening questionnaire multiple times on our website, either for themselves or for other relatives and friends. This was to allow for possible spillover effects, for example, if a user, after completion of the screening questionnaire, re-did the screening for another person. This, however, also implies that the same person could fill out the screening questionnaire multiple times with different information, for example, to check for related changes in the obtained diabetes risk score. The individual link id together with the IP address and browser information, however, allowed us to identify repeated survey questionnaires that were completed from the same device. We therefore construct a data sample in which we drop the observations stemming from repeated questionnaires, i.e., for each link id × IP address combination we keep only the first completed observation in our sample. We use this first observation based on the assumption that a person filling out the questionnaire multiple times would do so first for him- or herself and only afterward for another person. Similarly, we assume that if it was filled out multiple times simply out of curiosity, the respondent would enter the true data the first time and hypothetical data only afterward. This procedure led to a reduction from 1533 completed questionnaires (with duplicates) to an individual sample containing the 1469 completed screening questionnaires presented in the summary statistics.

Table 2 presents the summary statistics of the completed screening questionnaires for the main sample. Summary statistics, including the information for all started questionnaires and for the sample of completed questionnaires including any duplicates are presented in Supplementary Tables 4 and 5.

**Table 2:** Summary statistics of completed screening questionnaires

	(1)	(2)	(3)	(4)
	Mean	SD	Min	Max
Age distribution				
*Below 45*	0.32		0	1
*45–54*	0.47		0	1
*55–64*	0.16		0	1
*Above 65*	0.05		0	1
Female	0.49		0	1
Ever had high blood glucose	0.50		0	1
Ever diagnosed with high blood pressure	0.33		0	1
Family member with diagnosed diabetes	0.54		0	1
Weight	69.28	17.10	33	185
Height	162.43	7.84	140	195
BMI	26.15	5.61	11	70
Daily physical activity	0.60		0	1
Smoking				
*Never smoked*	0.66		0	1
*Stopped smoking*	0.20		0	1
*Currently smoking*	0.14		0	1
Daily fruit consumption	0.45		0	1
Daily sweet beverages consumption	0.30		0	1
Risk score	6.37	2.57	0	14
*Low risk*	0.14		0	1
*Medium risk*	0.25		0	1
*High risk*	0.61		0	1
Provided e-mail address	0.14		0	1
Number of observations	1469			

Table 2 displays the summary statistics of the completed screening questionnaires for the main sample without repeated answers.

The greatest proportion of users completing the risk screening questionnaire on our campaign website were in the 45–54 age group, the average BMI was about 26 and the users had on average a high diabetes risk with a risk score of 6.4. Sixty-one percent of them were found to be at high risk of diabetes, indicating that we were indeed able to reach out to persons who could benefit from such a self-screening. Men and women are almost equally represented. Half of the respondents report ever having been told that they have high blood sugar levels and one-third have ever been diagnosed with high blood pressure levels. In terms of smoking, 34% of participants report being ever-smokers, (i.e., either currently smoking or smoking previously but have now stopped). This average smoking rate, however, obscures a strong gender heterogeneity, with 8% of women and 57% of men in our sample being ever-smokers; a trend that is also well in line with the tobacco consumption pattern in Indonesia observed in the Indonesian Basic Health Research (RISKESDAS^48^, with 3.2% female and 65% male ever-smokers, respectively, for the total Indonesian population above the age of 10). Sixty percent of the respondents report doing at least 30 minutes of physical activity per day, while only 45% report consuming fruit or vegetables on a daily basis. Thirty percent of the respondents report consuming sugary beverages every day.

**Fig. 1:**
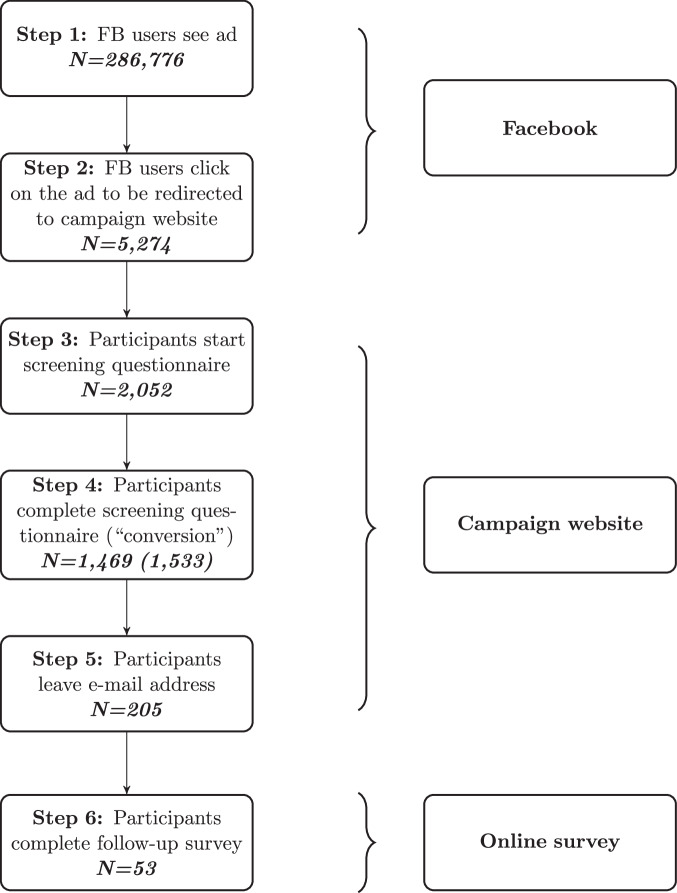
Workflow of the experiment. The number 1533 in parentheses at Step 4 refers to the number of completed questionnaires when duplicated questionnaires are also counted.

The summary statistics of the started screening questionnaires (Supplementary Table 4) reveal that a large share of survey starters dropped out after the first question (9%) and another large share before the question about participants’ weight and height (10%). Overall, 75% of started screening questionnaires were completed. Of all completers, 205 (14%) left their e-mail address to be contacted for further study activities. We sent a follow-up survey to this sub-sample six weeks after the end of the campaign. The full workflow and the number of observations at each step are presented in Fig. 1.

### Results from the follow-up survey

Of the 205 participants who left their e-mail addresses and agreed to be re-contacted for further research activities, 53 participated in the follow-up survey. The primary aim of the follow-up survey was to elicit whether individuals with a high risk of diabetes complied with the recommendation they received to schedule an appointment in a primary healthcare facility or with their physician to undergo a blood test for diabetes. Also, if they reported not planning to schedule an appointment, we were interested in the reasons. Of the 53 individuals participating in this survey, 32 (60%) had received a high-risk score in the screening, 15 (28)% a medium-risk score, and 6 (11%) a low-risk score. Obviously, we must assume that the group of respondents is not necessarily representative of the overall sample of 1469 individuals that participated in the screening, as survey participation was voluntary. However, when comparing their observable characteristics with those of the overall sample we did not find any statistically significant differences in their characteristics, as displayed in Supplementary Table 6. The power of these tests is of course limited, given the small sample size, but even the absolute size of the differences is in most cases surprisingly small. Moreover, we cannot detect any selection in terms of the ad the individual was exposed to (Supplementary Table 7), i.e., we do not find any significant effects of the different ads or the final risk score on the probability of participating in the follow-up survey.

We asked those individuals who either were at high risk according to their screening results or who mentioned remembering that they had a high risk about their plans for a professional appointment (n = 35). Of those individuals, 12 (34%) reported that they had already been aware that they had diabetes and hence no further professional test was needed, 13 (37%) reported that they did not plan to schedule a professional appointment, and 10 (28%) reported that they had already scheduled an appointment after participating in our screening or that they intended to do so in the next month (Supplementary Fig. 1). Hence, almost one-third of those deemed to be at high risk, corresponding to 43% of those who were unaware of their disease status, seem to comply with the recommendation to undergo a professional blood test for diabetes. If we extrapolate this share to the full sample, it amounts to 250 complying individuals at high risk. These numbers suggest that the campaign not only attracted individuals who were already aware that they had diabetes but that it also reached a substantial share of individuals at high risk of diabetes who were not aware of their status.

To account for a potential desirability bias in our survey, i.e., individuals simply reporting complying with the received recommendation because they expected this to be the socially desirable answer, we randomized two different framings of the same question. One highlighted the importance of scheduling a professional appointment given the possible severe health consequences of diabetes, the other implied that the time that had passed since the screening was probably too short to already have scheduled a meeting (the exact framings are shown in Supplementary Material 3). Whereas the first framing should increase the psychological cost of admitting to not having made an appointment, the second framing makes it psychologically rather easy to admit to not having made an appointment. If both framings lead to a comparable share of respondents who report having made an appointment, we can interpret this as evidence that a desirability bias is not at work. Indeed, we do not find any significant differences in the response pattern to the questions, which increases our trust in the reported answers (Supplementary Table 8).

Individuals reporting not intending to schedule an appointment for a professional blood test were further asked for the main reasons keeping them from doing so (Supplementary Fig. 2). More than half of the respondents answered being afraid of the possible costs of such a test. Given the small sample size for this question, the results have to be interpreted carefully. Yet, since preventive health care visits, including tests for chronic diseases, are free of charge for those covered by the JKN national health insurance scheme (which around 80% in our sample are), a potentially promising strategy to increase screening rates could be to distribute detailed information about the services covered in the scheme.

### Ad performance

Next to the assessment of the outreach and engagement with our campaign, we were interested in which ad design and framing would be most effective in creating clicks and conversions (completed screening questionnaires). In particular, we were interested in whether the two loss-framed ads would outperform the more neutrally framed ads (see the Methods section for the different designs). To assess ad performance, we estimate the following logistic regression models:

1 $$P(Link\,clic{k}_{i}=1| A{d}_{i}^{j},\,{{\bf{Z}}}_{i})=\lambda \left({\beta }_{0}+\mathop{\sum }\limits_{j=1}^{4}{\beta }_{j}A{d}_{i}^{j}+\beta {{\bf{Z}}}_{i}+{u}_{i}\right)$$

and

2 $$P(Conversio{n}_{i}=1| A{d}_{i}^{j},\,{{\bf{Z}}}_{i})=\lambda \left({\delta }_{0}+\mathop{\sum }\limits_{j=1}^{4}{\delta }_{j}A{d}_{i}^{j}+\delta {{\bf{Z}}}_{i}+{e}_{i}\right),$$

where *λ* is the logistic function, $\mathop{\sum }\nolimits_{j = 1}^{4}A{d}_{i}^{j}$ is a set of four dummy variables that are equal to one whenever person *i* saw ad *j* (the ad “family” serves as the reference group), **Z**_*i*_ is a vector of control variables (age, gender, region), and *u*_*i*_ (*e*_*i*_) is the error term. Note that the coefficients *β*_*j*_ and *δ*_*j*_ can be interpreted as causal effects since the ads were randomly assigned to Facebook users. Additionally, we investigate the effects separately by gender, since previous empirical evidence suggests that the effects of framing and information differ significantly for men and women^49–52^.

**Fig. 2:**
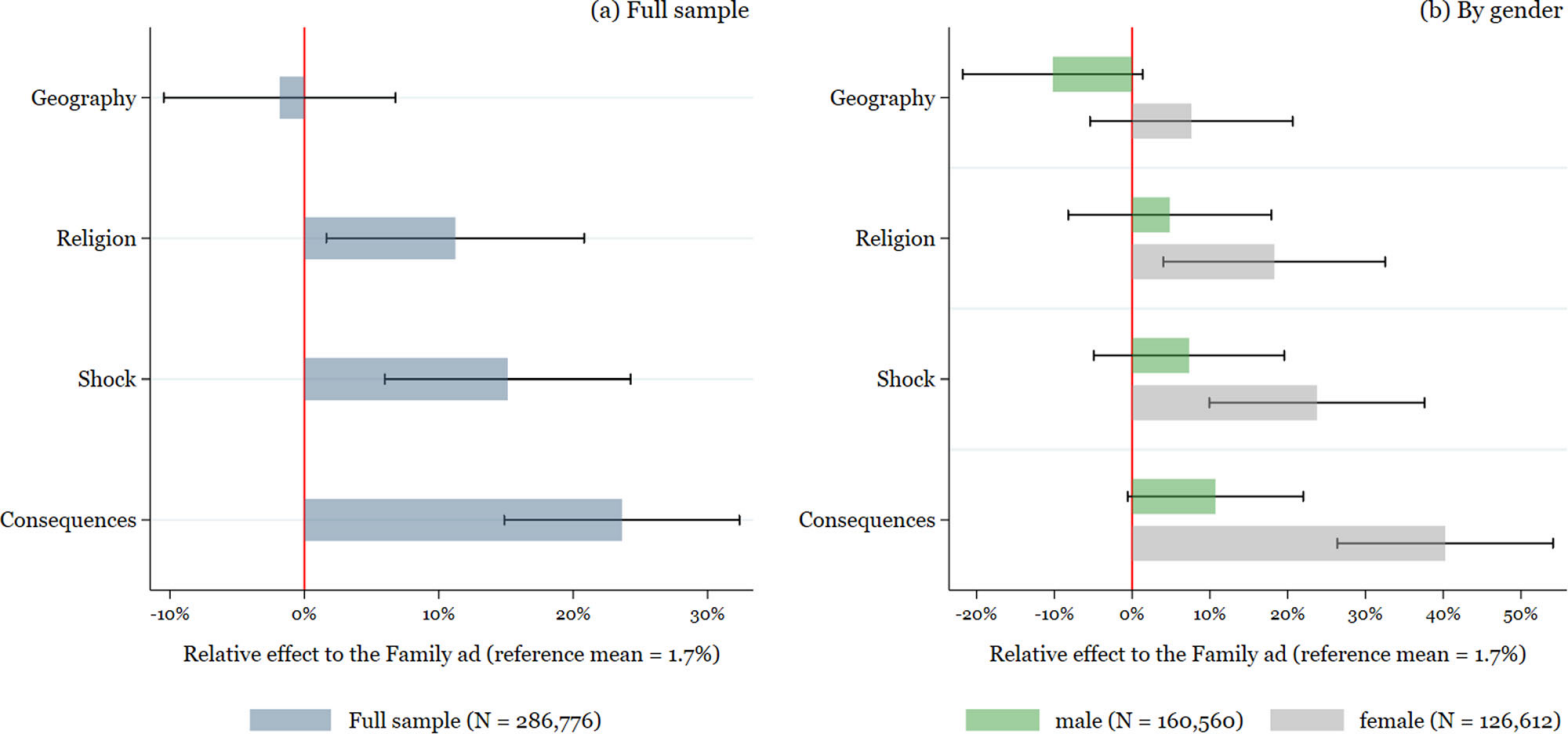
Ad effectiveness for link clicks. Figure 2 shows the effectiveness of the different ads in terms of link clicks for **a** the full sample and **b** by gender. The effects are presented as relative effect to the “family ad'', which serves as a reference category. Black whiskers present the 95% confidence intervals.

**Fig. 3:**
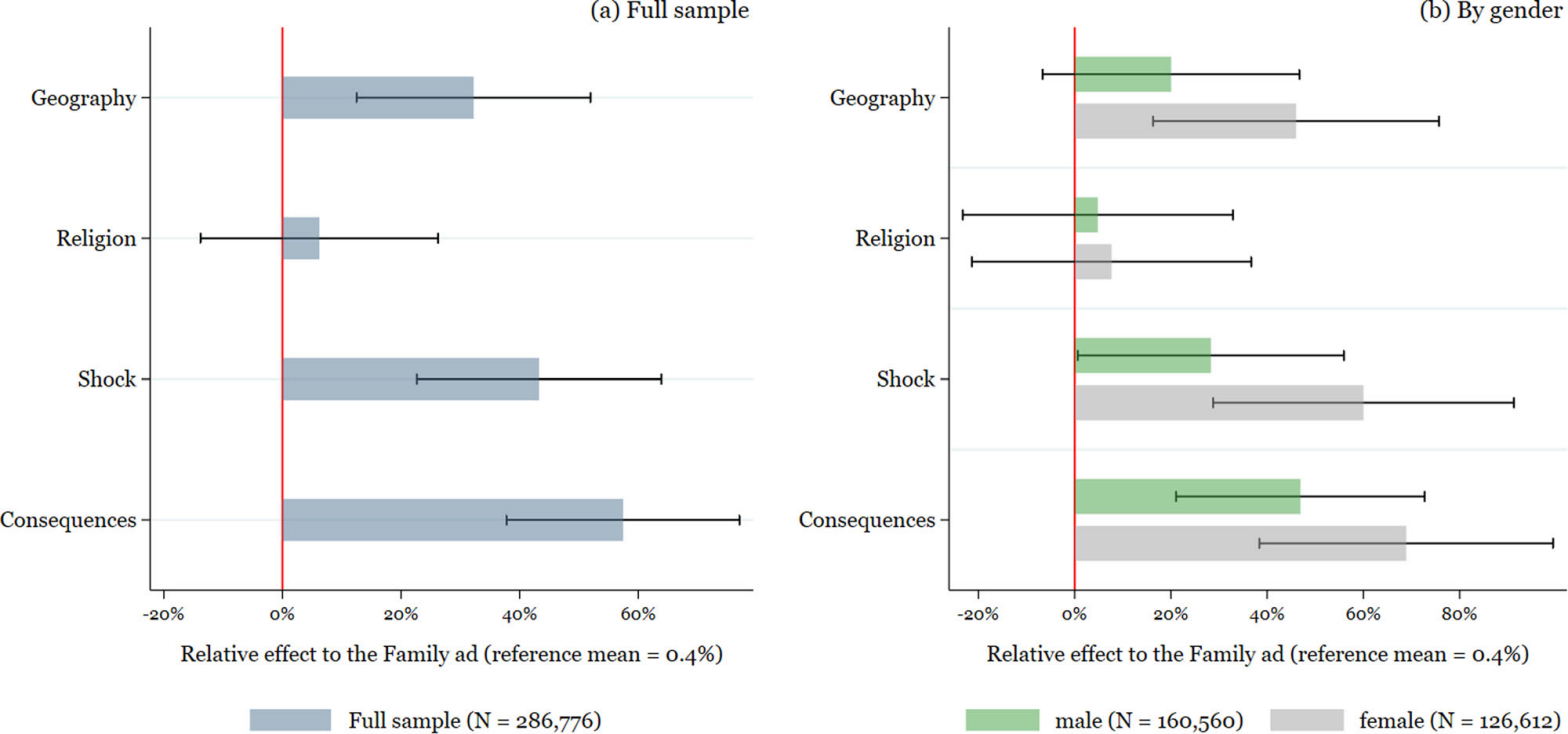
Ad effectiveness for conversions. Figure 3 shows the effectiveness of the different ads in terms of conversions for **a** the full sample and **b** by gender. The effects are presented as relative effects to the “family ad'', which serves as reference category. Black whiskers present the 95% confidence intervals.

Figures 2 and 3 together with Supplementary Tables 9 and 10 in Supplementary Material 4 show the results for link clicks and conversions for the total sample and separately for men and women. Figures 2 and 3 show the relative increases in comparison to the “family” ad, which implies a reference click-to-reach-ratio of 1.7% and a reference conversion-to-reach-ratio of 0.4%. Supplementary Tables 9 and 10 display the regression coefficients and marginal effects (with and without controls and by gender) from the logit model, together with the *p*-values of pairwise Wald tests for the different coefficients.

Graph (a) for the full sample in Fig. 2 shows that we can establish a clear hierarchy in terms of ad effectiveness for generating link clicks, with the two loss-framed ads clearly outperforming the ads “family” and “geography”. Only the effect of the “religion” ad is not statistically different from that of the “shock” ad. The performance of the “consequences” ad is somewhat larger than that of the “shock” ad, yet this difference is only significant at the 10% level (see also Supplementary Table 9).

In terms of the effect size, a user seeing one of the two loss-framed ads “shock” or “consequences” was 15% and 23%, respectively, more likely to click on the ad compared to someone who saw the least performing “family” ad. In absolute terms, this implies an increase to a click-to-reach-ratio of 1.9% and 2.1%. Those seeing the “shock” or “consequences” ads were also 3% and 11% more likely to click on the ads in comparison to the “religion” ad, though the differential effect between the “shock” and “religion” ads is not statistically significant. The magnitudes of these effects are comparable to those found in a study with a similar set-up, also based on Facebook’s A/B split function: Tjaden et al.^12^ test several ads to increase Covid-19 vaccination rates in Germany and vary the pictured messenger (doctor, governmental representative, religious leader). They report an increase between 20% and 40% in clicks of the best-performing versus other ads.

Differentiating the ads’ effectiveness by gender (Graph (b)), however, shows that the effectiveness of the “consequences” and “shock” ads in terms of link clicks seems to be driven by women, whereas men reacted to all ads in a rather similar manner. In fact, while the effect is still the largest for the two loss-framed ads in qualitative terms, we cannot reject the hypothesis of equal performance of all five ads for the male audience.

Turning to conversions, Fig. 3, Graph (a) shows a slightly different picture. While the “consequences” ad is again the best-performing ad in generating conversions (significantly different from all but the “shock” ad), the performance of the “religion” ad, which was the one that came closest to the performance of the loss-framed ads in terms of creating link clicks, is no longer significantly different from the least performing “family” ad. This might be a sign that the “religion” ad did not sufficiently relate to the topic of diabetes and viewers of the ad did not proceed to the screening once they realized that the website did not contain religious content.

In contrast, the “geography” ad is significantly more effective than the “family” and “religion” ads and equally effective as the “shock” ad in generating finalized risk screening tests. Differentiating by gender (Graph (b)) reveals, however, that the effectiveness of the “geography” ad is again solely due to female users. For men, responsiveness to the “consequences” ad was greatest and the ad performed significantly better compared to all other ads with the exception of the “shock” ad (*p*-value 0.188).

The effect magnitudes are somewhat larger than those for link clicks when comparing the best-performing ad against the others: an individual exposed to the consequences ad was 57%, 48%, 19% and 10% more likely to complete the self-screening than someone seeing the family, religion, geography or shocking ad, respectively.

Women were also more likely overall (+25%) to complete a screening questionnaire conditional on seeing any of the ads compared to their male counterparts. Yet, given that the number of women seeing an ad on Facebook was lower in absolute terms (since there are generally fewer female Facebook users than male users in Indonesia^45^), the sample of completed questionnaires is balanced in the gender distribution. Although the oldest age group (65+) was more likely to click on the ads than users below the age of 45, they are about equally likely to complete the questionnaire as the youngest age group, which is driven by a higher attrition rate in the oldest age group. Specifically, when we regress the probability of attrition on participants’ characteristics (conditional on having started the screening questionnaire), we find that elderly respondents above the age of 65 were 34 percentage points more likely to drop out in the course of the questionnaire compared to the youngest age groups. This effect is larger for older men, though not statistically different from the effect for older women (results shown in Supplementary Table 11).

Overall, we can confirm the hypothesis that an ad with a loss-framed perspective, i.e., highlighting the adverse health consequences of diabetes, performs significantly better than ads referring to the family, religion, or local prevalence rates. Only the second loss-framed and “shocking” ad comes close to the performance of the “consequences” ad in our health awareness campaign. Hence, an online diabetes awareness campaign focusing on the health consequences of diabetes can be an effective tool to induce diabetes self-screenings. When we assess whether the diabetes risk level of the screening completers differs in relation to the ad they saw, we find that those who saw one of the loss-framed ads had a risk score that was on average higher by 0.28 (*p*-value 0.039) than the score of those who saw one of the other three ads. This supports the hypothesis by Rothman et al.^31,32^ by showing that those who do indeed have a higher diabetes risk, and might also perceive it as such, were more responsive to the loss-framing ads than someone with a lower risk.

Our campaign also shows that the content and framing of the ads is particularly important when targeting women. Women reacted more differentially to the different ads, whereas men responded to the ads more equally, especially for the outcome of link clicks. Yet, also for men, the “consequences” ad performed significantly better than the “family”, “geography” and “religion” ads for the conversion outcome, indicating that the loss-perspective was successful in engaging men in the actual self-screening activity.

While such gender-heterogeneous responses are in line with previous research highlighting the moderating effect of gender in loss- versus gain-framing experiments e.g.,^49–52^, we must refrain from a more extensive analysis of the drivers of this effect, simply due to data limitations. We did not collect any information on underlying characteristics that could explain such differential behavior. Yet, the literature suggests that gender-differences in risk perceptions^49^, avoidance orientation^50^ or trust^41^ can shape these gender-specific responses. Also, we did not explicitly test loss- versus gain-framing but rather loss-focused versus differently focused ads, which limits the comparability of our results with more precise gain- versus loss-framed campaigns. Nevertheless, our results provide important insights into the question of what type of ads can effectively be used to enhance preventive health behavior and how responsiveness differs between men and women.

### Comparison of the sample and benchmark populations

A valid concern that might arise at this point is that we were only able to reach out to a particular population group with our Facebook campaign. While the distribution of the ads was random conditional on being in the pre-specified target group, the actual selection into completing the screening questionnaire is endogenous, and hence the results concerning the effectiveness of our campaign might not to be generalized to other population groups. To investigate the importance of such selection effects, we compare our sample of participants who completed the screening questionnaire with the universe of people who met our eligibility criteria in Jakarta and Yogyakarta. This comparison is presented in detail in Supplementary Material 5 and Supplementary Table 12. It suggests that the sample generated by our experiment is slightly skewed toward the 45-55 age group and to those who seem to be significantly more at risk of having or developing diabetes compared to the total population above the age of 35 in Jakarta and Yogyakarta. We interpret this self-selection as an indication that our campaign was very effective in reaching out to people at high risk who could potentially benefit from such online screening. Since we also showed above in the results of the follow-up survey that only one-third of the individuals who were found to have a high risk and that self-selected into the follow-up survey had already been aware that they have diabetes, we deem this as evidence that our campaign was indeed able to reach out to a large number of individuals who were unaware of their high risk and that our campaign was able to effectively engage them in the diabetes self-screening.

### Cost-effectiveness

Having identified that ads focusing on the detrimental health consequences of diabetes can be a particularly well-suited approach to encourage diabetes risk screening among those with a comparably high diabetes risk, we are now interested in the cost-effectiveness of such an online campaign. We analyze the cost-effectiveness of our Facebook health campaign under the assumption that it would be scaled-up to a one-year health campaign across the whole island of Java. This implies a target population of about 25 million Facebook users above the age of 35. We perform a simple cost-effectiveness calculation based on the cost and effectiveness parameters derived from our study and enrich them with a repeated decision-tree model. The final cost parameter of interest is the cost per newly diagnosed person.

The assumptions, results, and sensitivity analysis of the cost-effectiveness analysis are presented in Supplementary Material 6 (Supplementary Tables 13 and 14 and Supplementary Fig. 3). We show that the hypothetical up-scaling of the campaign to the whole of Java over the period of one year could lead to about 1.7 million users participating in the online screening, of whom about 250,000 would continue with the professional follow-up screening, and finally to the diagnosis of almost 170,000 previously undetected diabetes cases. This corresponds to an increase from 25% to 29% of diagnosed cases relative to all cases, i.e., an increase of 16%. While the share might still seem small, the absolute number is large, especially in light of the low cost and low effort needed to implement an online health campaign. This low cost is further confirmed when we look at the total cost of the proposed intervention (including the professional follow-up screening), which is slightly higher than US*$*1.5 million. Dividing the total cost by the 170,000 newly diagnosed cases, the cost of detecting one more previously undiagnosed person amounts to approximately US*$*9 (with a lower bound of US*$*5.20 in a best-case scenario and an upper bound of US*$*37 in a worst-case scenario).

Contrasting these amounts to the cost of long-term diabetes care in Indonesia suggests a large cost-saving potential. Hidayat et al.^53^ estimate the direct medical costs for a patient in the Indonesian healthcare system with severe diabetes health consequences at US*$*930 per person per year, whereas a patient without severe diabetes consequences costs the healthcare system only US*$*420. Under the premise that early diagnosis reduces the probability of severe diabetic health consequences, an online diabetes health campaign offers the possibility of reducing healthcare expenditures in the long term. Further, the cost per detected case is lower in comparison to other screening strategies, for example, screening with a similar diabetes risk questionnaire during annual health check-ups in Thailand (~US*$*30 per detected case, counting only direct medical cost)^54^.

## Discussion

NCDs are the leading cause of death worldwide. In LMICs, the health and economic burden due to NCDs is rising rapidly and innovative solutions to increase screening activities and encourage healthy lifestyles could counteract this problem. Public health campaigns can help to increase awareness of NCDs and encourage populations at risk to change unhealthy lifestyles, inform them about important preventive health measures such as screening, ensure adequate treatment in the event of a positive diagnosis, and thereby reduce health care costs and productivity losses in the long run.

We show that using social media platforms, such as Facebook, for such health campaigns sets out new opportunities to increase awareness and screening for diabetes in LMICs. Such campaigns can generate high exposure and engagement rates at very low cost. With our campaign, we were able to reach out to almost 300,000 individuals in only three weeks and with a budget of less than US*$*1100. More than 1400 individuals completed the offered online diabetes risk screening on our campaign website, implying a cost of less than US*$*0.75 per person screened in that way. We also relied on insights from psychology and assessed whether such a campaign should rely on ads with a focus on a loss-framed or shocking perspective to effectively induce preventive health screenings. Our randomized experiment shows that this is indeed a promising approach and that ads focusing on the adverse health consequences of diabetes are most effective in nudging viewers to click on the ads and to carry out a diabetes self-screening. In particular, we find that an ad highlighting the risk of losing eyesight or developing heart- and kidney diseases as a consequence of diabetes outperformed all other ads in the number of link clicks and completed screening questionnaires. Only the second loss-framed ad, which focused on the fact that diabetes can result in death, came near the performance of the “consequences” ad. Yet, this framing effect was more pronounced for the female sample in our study. Men responded more equally also to other ads. These gender differences should be considered by policymakers aiming to design an effective public health campaign.

We also find that such a campaign is especially well-suited for reaching out to the population in the 45–55 age range. This is an encouraging finding, given that the risk of diabetes increases after the age of 45 and a diagnosis of elevated blood sugar at this age offers the opportunity for early treatment to prevent further adverse health consequences.

However, while we can establish that loss-framed or more shocking ads are more effective in terms of creating link clicks and completed self-screenings, it is beyond the scope of our study to assess whether such negatively framed ads could have longer-term negative consequences. A potential adverse effect could for example arise if individuals exposed to the loss-framed ads would engage in information avoidance. In the context of our study, we can show that those individuals being exposed to the loss-framed ads were more likely to participate in the self-screening and equally likely to participate in the follow-up survey, indicating that they did not engage in information avoidance in the short term. Yet, we cannot rule out that long-term health behavior after having received a high-risk result in the self-screening could be adversely affected by the prospect of negative health consequences. Moreover, while shocking contents work well in social media networks to go viral, such content could also induce anxiety or trigger mental health consequences. A recent study in the context of Covid-19^55^, for example, shows that loss-framed ads increased anxiety levels. Together with the fact that a diabetes diagnosis can lead to diabetes distress^56^ and affected individuals are at increased risk for mental health disorders^57^, our results call for further research in terms of longer-term consequences of using loss-framed ads in public health campaigns, especially when implemented at scale.

A remaining limitation of our study is that our measure of compliance with the received recommendation to visit a physician or the report of an existing diagnosis is self-reported. Even though we control for social desirability bias, we are limited in our ability to measure whether individuals claiming to have scheduled an appointment indeed follow through with the professional screening, or whether an individual indeed was already diagnosed with diabetes before. This leaves ample room for future studies in which actual compliance rates are being measured. This could be done, for example, by cooperating directly with local health centers that verify whether a person was referred via an online campaign (e.g., via a referral voucher). Moreover, to confirm the self-reported diabetes diagnoses, it would be interesting to set up a study aiming to verify existing diagnoses through medical records. Yet, privacy concerns and data protection rules pose a substantial hurdle for such a study design.

While we run our campaign in Indonesia, many other middle-income countries are equally experiencing a rapidly increasing diabetes burden and have high social media usage rates. This suggests that the insights from our campaign and study should not only be transferable to other countries in Southeast Asia but also to countries such as India, Brazil, Mexico, and Pakistan.

Overall, our study suggests that a health awareness campaign implemented on the social media platform Facebook is a useful tool to increase awareness of and (self-)screenings for diabetes, and loss-framed ads work particularly well. Policymakers in Indonesia and comparable countries should consider using such social media health campaigns as an innovative tool to address the increasing diabetes burden.

## Methods

### Campaign and ad design

From March 15 until April 5, 2022, we ran a diabetes health campaign on Facebook, targeting Indonesian Facebook users in Jakarta and Yogyakarta – the two cities with the highest diabetes rates in Indonesia^48^. In Indonesia’s urban areas, which also have higher diabetes prevalence rates than rural areas, internet penetration rates and usage of social media platforms are high. As of January 2022, the internet penetration rate in Indonesia stood at 74%, with 94% of all users accessing the internet via smartphones. Around 190 million Indonesians are active social media users, of which 130-135 million are active Facebook users, according to the audience size to be reached with Facebook’s advertising tool^58,59^.

We implemented the campaign via Facebook’s advertisement function which permits the distribution of self-designed ads to Facebook users while using specific demographic and geographic targeting criteria. This advertisement tool was originally developed for businesses to boost their customer base and increase sales, but it is also increasingly used by scientific researchers to recruit survey participants^43,60–63^. While using the tool for the recruitment of survey participants is indisputably practical, it also offers an even more sophisticated and scientifically valuable function that allows researchers to implement randomized controlled trials. Facebook’s A/B split test allows for a random distribution of two or more ads to evenly split and statistically comparable audiences to test which ad performs best in terms of a pre-specified campaign target^64^. The ads can thus differ in their design or placement, depending on which variable is being tested. This A/B test design also ensures that the same budget is allocated to each ad and hence avoids Facebook’s algorithm determining the budget allocation, something which could generate unbalanced Facebook user exposure rates across ads.

**Fig. 4:**
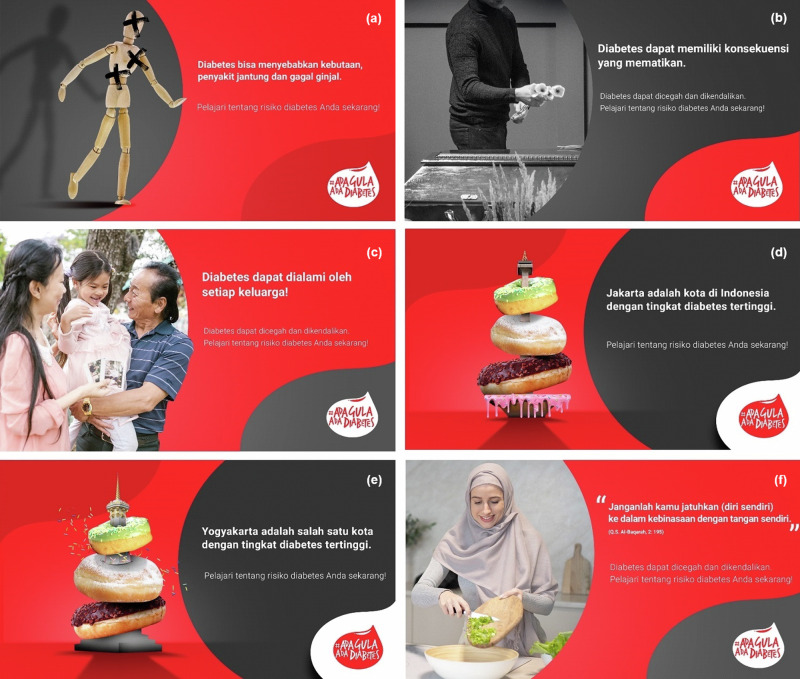
Ad design. **a** Diabetes consequences – Diabetes can cause blindness, heart diseases, and kidney failure. Learn about your diabetes risk now! **b** Shock – Diabetes can have deadly consequences. Diabetes can be prevented and controlled. Learn about your diabetes risk now! **c** Family – Diabetes can affect every family. Diabetes can be prevented and controlled. Learn about your diabetes risk now! **d** Geography (Jakarta) – Jakarta is the city with the highest diabetes prevalence rate in Indonesia. Learn about your diabetes risk now! (**e**) Geography (Yogyakarta) – Yogyakarta is one of the cities with the highest diabetes prevalence rates. Learn about your diabetes risk now! **f** Religion – “and do not throw [yourselves] with your [own] hands into destruction” (Q.S. Al-Baqarah, 2:195). Diabetes can be prevented and controlled. Learn about your diabetes risk now!

We designed five different ads, two of which took on a loss-framed and rather disquieting perspective, with the remaining three referring to the family, religion, and the local diabetes prevalence rate. The two loss-framed ads were entitled “diabetes consequences” and “shock”. The non-loss-framed ads were entitled “family”, “religion” and “geography”. These non-loss-framed ads were inspired by different strands of the literature that link religion and health^65^, family and health^66^, and information about local health conditions and health behavior^67^. While this design does not allow us to infer the effects of loss- versus gain-framing (since we do not include a specifically gain-framed ad), it allows us to compare the effect of loss-framed ads with ads that rely on different psychological channels that have been shown to affect health-related behavior. The ads and their displayed message are described in more detail below and presented in Fig. 4.

1. Consequences: The consequences ad contained a statement about the possible health consequences of diabetes, including blindness, kidney- and heart diseases. The graphic showed a wooden mannequin on which the body parts that can be affected by diabetes were marked with a black cross.
2. Shock: The shocking ad pictured a man in front of a coffin and contained the message that diabetes can have deadly consequences.
3. Family: The family ad pictured three generations of an Indonesian family and contained the message that every family can be affected by diabetes.
4. Geography: One geography ad was designed for each of the two regions in our study (Jakarta and Yogyakarta). The graphics showed a landmark of each of the two cities (the National Monument in Jakarta and the Yogyakarta Monument in Yogyakarta, respectively) covered in sweets. The message referred to the local prevalence rate of diabetes in each of the regions.
5. Religion: The religion ad presented an Indonesian woman in hijab cooking and contained a statement from the Quran that conveyed the message that one should not live a potentially self-harming life.

In addition to the messages outlined above, each ad carried the statement “Learn about your diabetes risk now” (“Pelajari tentang risiko diabetes Anda sekarang”) to encourage the ad viewers to click on the ad and visit the campaign website on which they could conduct the risk screening test. Technically, we ran two different campaigns, one for each of the targeted regions, and then pooled the data for the analysis. Each ad received an equal budget of US*$*5 per day, summing to a total daily budget of US*$*50 for both cities. In terms of the target population, we restricted the audience demographically to Facebook users above the age of 35 and geographically to users living in either Jakarta or Yogyakarta.

**Fig. 5:**
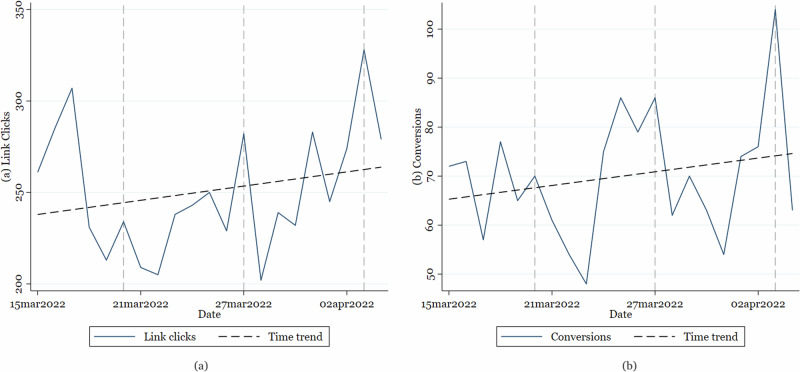
Link clicks and conversions over time. Figure 5 displays the time trend in **a** daily link clicks and **b** daily conversions (i.e., completed screening questionnaires). Vertical gray dashed lines indicate Sundays.

The campaign objective was chosen to optimize “conversions”, with conversion programmed to be equal to completion of the screening questionnaire. Setting “conversions” as the campaign objective (instead of the other two possibilities “awareness” or “consideration”) allowed us to focus on possible screening questionnaire completers who would thus gain from the campaign, while simultaneously preventing showing the ads to seemingly uninterested users. This conversion objective required the generation of a so-called Facebook Pixel code, which had to be embedded in the code of the website to which the ad viewers were redirected. Facebook could then use this Pixel to track user actions taking place on our website and optimize accordingly. This implies that after a learning phase, Facebook’s algorithm aimed to show the ads to individuals more likely to click on the ads and to complete the screening questionnaire, based on the characteristics of earlier completers. The success of the algorithm is confirmed by the positive trend in the number of daily clicks and conversions over time as presented in Fig. 5. After a first peak in link clicks, most likely driven by immediate reactions from viewers always responding to such ads, the learning phase sets in and translates into a positive trend in clicks and conversions. While this internal algorithm exaggerates a selection bias per ad if the conversion objective is used in regular campaigns^68^, the use of the A/B split test ensured that, conditional on being in the target audience, the ad version the user saw was random. This randomization procedure allowed us to compare the different ads based on their effectiveness in generating clicks and conversions, i.e., completed screening questionnaires.

### Campaign website

After clicking on one of the ads in Facebook, individuals were redirected to the landing page of the campaign website. Before being able to browse further on the website, the participants were informed about our privacy policy and that data generated on the website were used for an academic study. For both, they had to indicate their informed consent. Individuals were then offered the opportunity to complete a diabetes risk screening questionnaire on this website similar to the diabetes risk test of the American Diabetes Association and the diabetes FINDRISC (Finnish Diabetes Risk Score) screening test. The questionnaire version we used is an adapted and translated version specifically for the Indonesian population. The original FINDRISC questionnaire was developed to identify individuals at risk of diabetes using a Finnish population sample^69^. Since then, the questionnaire has been evaluated and validated many times and has been adjusted to different populations and country samples^70–73^. The original diabetes risk test of the American Diabetes Association dates back to 1993 and has likewise been adapted multiple times^74^. The version we used is based on the diabetes risk test of the American Diabetes Association^75,76^, the ModAsian FINDRISC for Asia, the FINDRISC Bahasa Indonesia^77^, and the Malay version of the American Diabetes Association diabetes risk test^74^. It consisted of eleven questions which could be answered in approximately 90 seconds. Based on the individual answers, a risk score between 0 and 16 points was calculated and participants received an assessment of their personal risk rated as low risk (0-3 points), medium risk (4-5 points), or high risk (6 or more points). Additionally, the assessment contained recommendations on how to keep the risk low, how the diabetes risk can be reduced and to visit a health center or a physician if the risk score was too high.

The website also included a page with factual information on diabetes in Indonesia, including the distribution of prevalence rates across the country, behavioral risk factors, as well as information about how diabetes can be diagnosed and how it can be treated. Furthermore, we provided detailed information about the institutions involved in the research activities, the aim of the campaign, and the notification that the campaign was purely educational and could not replace a professional health visit or screening. We also asked participants to leave their e-mail addresses so that they could get follow-up information and continue to be involved in the study.

### Follow-up survey

Six weeks after the end of the campaign we sent a follow-up survey to all these addresses to elicit information about actual compliance with the recommendations received. Since providing the e-mail address was voluntary and hence the sub-sample of respondents was subject to a potential self-selection bias, we provide a description of the sample that completed this follow-up survey and contrast it with the profile of the entire sample (see Results section). In this follow-up survey, we asked the respondents about their plans to comply with the received recommendations. Specifically, we asked whether they plan to schedule a professional medical screening (or have already done so), if yes, when and where they planned to go and if no, what their reasons were for not doing so. We also asked several questions about diabetes risk factors, symptoms, and health consequences, whether the respondent had health insurance, whether this was the first time they had conducted a diabetes risk test, whether they had already been diagnosed with diabetes, and whether they were currently on medication.

### IRB approval and RCT registration

This study received ethical approval from the University of Passau Research Ethics Committee (15.03.2022, IRB Approval Number I-07.5090/2022). Informed consent was obtained from all participants who browsed our website. Informed consent for the experiment on Facebook is covered by Facebook’s data use policy. Identifiable images relating to persons in our ads are no patients and no written consent was required since ads were designed by ourselves with pictures taken from openly accessible stocks with license-free images. The study was pre-registered at the AEA RCT Registry (0008781, 10.1257/rct.8781). In the final manuscript/study, we deviated in some features from our initial analysis plan, partly for technical reasons, and marginally adjusted our hypotheses after the pilot study. These changes are explained in detail in an appendix to our pre-analysis plan (downloadable under the same registration number). The study was conducted without any support from or connection to Facebook (Meta group) and Facebook had no access to the responses that were generated on our website or during the follow-up survey.

## Supplementary information

Supplemental material

## Data Availability

All data underlying this study are available from the authors upon request.

## Code availability

All codes underlying this study are available from the authors upon request.
