# Supplementary material for: Can social media encourage diabetes self-screenings? A randomized controlled trial with Indonesian Facebook users
Source: NPJ Digit Med. 2024 Sep 13;7:245. doi: 10.1038/s41746-024-01246-x (PMC11399376; doi:10.1038/s41746-024-01246-x)
Supplement: Supplementary file 1 — Supplemental material [file 41746_2024_1246_MOESM1_ESM.pdf]

Supplementary Material

This document contains the supplementary material for the article "Can social media encourage diabetes self-screenings? A randomized controlled trial with Indonesian Facebook users".

Supplementary Material 1: Screening questionnaire 2-3

Supplementary Material 2: Additional summary statistics 4-6

Supplementary Material 3: Additional tables and figures - follow-up survey 7-11

Supplementary Material 4: Additional tables and figures - ad heterogeneity 12-14

Supplementary Material 5: Comparison of the sample and benchmark populations 15-17

Supplementary Material 6: Cost-effectiveness 18-22

References 23

Supplementary Material 1: Screening questionnaire

Supplementary Table 1: Screening questionnaire and scoring system (English)

No.	Question	Alternatives	Score
1	How old are you?	Younger than 45	0
		45-55	1
		56-65	2
		Older than 65	3
2	What is your gender?	Female	0
		Male	1
3	Have you ever been found to have high blood sugar levels? (This could be, for example, in a health examination, during an illness or during pregnancy.)	Yes	3
		No	0
4	Have you ever been diagnosed with high blood pressure or are you taking any anti-hypertensive drugs?	No	0
		Yes	1
5	Do you have a mother, father, sister or brother who was diagnosed with diabetes?	No	0
		Yes	1
6	What is your weight? (in kg)		
7	What is your height? (in cm)		
	BMI calculated from 6 and 7	≤ 23	0
		23 – 27.5	1
		27.5 – 32.5	2
		≥ 32.5	3
8	Do you usually have at least 30 minutes of physical activity per day? (Physical activity means moderate or vigorous activity such as walking, swimming, badminton etc.)	Yes	0
		No	1
9	Do you smoke cigarettes?	No, I never smoked.	0
		No, I stopped smoking.	0
		Yes	1
10	Do you eat fruit or vegetables every day? (Please do not count fruit juices.)	Yes	0
		No	1
11	Do you drink sugary beverages every day? (Examples of these are soft drinks, sweetened tea or fruit juices with sugar.)	No	0
		Yes	1
**Scoring**			
≤ 3	**Low risk**		
4–5	**Medium risk**		
≥ 6	**High risk**		

Notes: Adapted from the American Diabetes Association[1,2], Lindstrom and Tuomilehto[3], Fauzi *et al.*[4], and Rokhman *et al.*[5]

Supplementary Table 2: Screening questionnaire and scoring system (Bahasa Indonesia)

No.	Question	Alternatives	Score
1	Berapa umur Anda?	Di bawah 45 tahun	0
		45-55 tahun	1
		56-65 tahun	2
		Di atas 65 tahun	3
2	Apa jenis kelamin Anda?	Perempuan	0
		Laki-laki	1
3	Apakah hasil pemeriksaan gula darah Anda pernah tinggi? (Contohnya saat tes kesehatan, ketika sakit, atau selama kehamilan.)	Ya	3
		Tidak	0
4	Apakah Anda pernah didiagnosis menderita tekanan darah tinggi atau sedang mengonsumsi obat antihipertensi?	Tidak	0
		Ya	1
5	Apakah Anda memiliki ibu, ayah, saudara perempuan atau saudara laki-laki yang menderita diabetes?	Tidak	0
		Ya	1
6	Berapa berat badan Anda? (dalam kilogram)		
7	Berapa badan tinggi Anda? (dalam sentimeter)		
	IMT berdasarkan 6 dan 7	≤ 23	0
		23 – 27.5	1
		27.5 – 32.5	2
		≥ 32.5	3
8	Apakah Anda biasa melakukan aktivitas fisik, minimal 30 menit per hari? (Aktivitas fisik yang dimaksud adalah aktivitas fisik sedang atau berat seperti berjalan kaki, berenang, bulu tangkis, dll.)	Ya	0
		Tidak	1
9	Apakah Anda merokok?	Tidak, saya tidak pernah merokok.	0
		Tidak, saya berhenti merokok.	0
		Ya	1
10	Seberapa sering Anda mengkonsumsi sayur atau buah? (Tidak termasuk jus buah.)	Ya	0
		Tidak	1
11	Seberapa sering Anda mengkonsumi minuman manis? (Contohnya adalah minuman ringan, minuman bersoda, teh manis, atau jus buah dengan gula.)	Tidak	0
		Ya	1
**Skor**			
≤ 3	**Risiko rendah**		
4–5	**Risiko sedang**		
≥ 6	**Risiko tinggi**		

Notes: Adapted from the American Diabetes Association[1,2], Lindstrom and Tuomilehto[3], Fauzi *et al.*[4], and Rokhman *et al.*[5]

Supplementary Material 2: Additional summary statistics

Supplementary Table 3: Age, gender and location distribution by ad

	(1) Full Sample	(2) Family	(3) Consequences	(4) Geography	(5) Religion	(6) Shock
Gender (1=male)	0.44	0.48	0.38	0.45	0.48	0.44
Below 45	0.48	0.46	0.50	0.50	0.44	0.46
45-54	0.34	0.34	0.33	0.33	0.37	0.35
55-64	0.11	0.13	0.11	0.10	0.12	0.12
65+	0.07	0.07	0.06	0.07	0.07	0.07
Location (1=Jakarta)	0.51	0.50	0.54	0.49	0.50	0.50
N	286,776	55,576	73,264	59,952	43,584	54,400

Notes: Supplementary Table 3 presents the age, gender and location distribution for the total sample and by ad. All variables are binary variables, hence, the summary statistics are shares of the respective variable.

Supplementary Table 4: Summary statistics of started screening questionnaires (with duplicates)

	(1) Mean	(2) SD	(3) Min	(4) Max	(5) Obs.
Age					2,052
Below 45	0.28				
45-54	0.46				
55-64	0.18				
Above 65	0.08				
Female	0.48				1,873
Ever had high blood glucose	0.50				1,810
Ever diagnosed with high blood pressure	0.34				1,794
Family member with diagnosed diabetes	0.53				1,777
Weight	69.30	16.87	33	185	1,461
Height	162.51	7.86	140	195	1,561
BMI	26.13	5.52	11	70	1,561
Daily physical activity	0.60				1,544
Smoking					1,541
Never smoked	0.65				
Stopped smoking	0.20				
Currently smoking	0.15				
Daily fruit consumption	0.45				1,539
Daily sweet beverages consumption	0.30				1,533
Risk score	6.34	2.58	0	14	1,533
Low risk	0.15				
Medium risk	0.25				
High risk	0.60				

Notes: Supplementary Table 4 presents the summary statistics for all questionniares that were started, with duplicates.

Supplementary Table 5: Summary statistics of completed screening questionnaires (with duplicates)

	(1) Mean	(2) SD	(3) Min	(4) Max	(5) Obs.
Age					1,533
Below 45	0.32				
45-54	0.46				
55-64	0.16				
Above 65	0.05				
Female	0.48				1,533
Ever had high blood glucose	0.49				1,533
Ever diagnosed with high blood pressure	0.33				1,533
Family member with diagnosed diabetes	0.54				1,533
Weight	69.31	16.95	33	185	1,533
Height	162.51	7.81	140	195	1,533
BMI	26.14	5.55	11	70	1,533
Daily physical activity	0.60				1,533
Smoking					1,533
Never smoked	0.65				
Stopped smoking	0.20				
Currently smoking	0.15				
Daily fruit consumption	0.45				1,533
Daily sweet beverages consumption	0.30				1,533
Risk score	6.34	2.58	0	14	1,533
Low risk	0.15				
Medium risk	0.25				
High risk	0.60				

Notes: Supplementary Table 5 presents the summary statistics for all questionniares that were completed, with duplicates.

Supplementary Material 3: Additional tables and figures - follow-up survey

1. Differences between full sample, e-mail providers and follow-up completers

Supplementary Table 6: Differences between full sample, e-mail providers and follow-up completers

	(1) Full Sample	(2) E-mail providers	(3) Follow-Up completers	(4) Difference (1)-(2)	(5) Difference (1)-(3)
Age distribution					
35-45[1]	0.32	0.21	0.23	0.12***	0.09
45-54	0.47	0.52	0.60	-0.06***	-0.14
55-64	0.16	0.20	0.11	-0.04***	0.05
Above 65	0.05	0.07	0.06	-0.02***	-0.00
Female	0.49	0.50	0.45	-0.02	0.04
Ever had high blood sugar levels	0.50	0.65	0.60	-0.18***	-0.10
Ever diagnosed with high blood pressure	0.33	0.33	0.36	0.01	-0.03
Family member with diagnosed diabetes	0.54	0.60	0.57	-0.06	-0.02
Weight	69.28	67.86	68.37	1.65	0.95
Height	162.43	161.73	162.53	0.82	-0.10
BMI	26.15	25.85	25.83	0.35	0.33
Daily physical activity	0.60	0.60	0.55	0.00	0.06
Smoking					
Never smoked[1]	0.65	0.62	0.63	0.01	0.04
Stopped smoking	0.20	0.20	0.19	-0.00	0.01
Currently smoking	0.14	0.15	0.19	-0.00	-0.05
Daily fruit consumption	0.45	0.46	0.43	-0.01	0.02
Daily sweet beverages consumption	0.30	0.30	0.38	0.01	-0.08
Risk score	6.37	6.99	6.91	-0.72***	-0.56
Low risk[1]	0.14	0.11	0.11	0.04	0.03
Medium risk	0.25	0.22	0.28	0.03	-0.04
High risk	0.61	0.67	0.60	-0.07	0.01
Joint orthogonality test vs. full sample				0.000	0.458
Joint orthogonality test vs. E-mail providers					0.681
N	1,469	205	53		

Notes: Supplementary Table 6 presents the results of the comparisons between the full sample of participants that completed the screening, the e-mail providers and the sample of participants that completed the follow-up survey. *** p<0.01, ** p<0.05, * p<0.1. [1]Statistical differences in the distribution of categorical variables (age groups, smoking status, risk groups) are assessed via Pearson's chi-squared test and significance stars are added to each category.

Supplementary Table 7: Email and follow-up survey response rates across ad designs and as a function of risk score

	(1)<br>E-mail<br>provision	(2)<br>E-mail<br>provision	(3)<br>Follow-up<br>participation	(4)<br>Follow-up<br>participation
**Reference: Family**				
Consequences	0.001		0.002	
	(0.029)		(0.016)	
Geography	0.003		0.029*	
	(0.031)		(0.017)	
Religion	0.041		0.005	
	(0.035)		(0.019)	
Shock	0.005		0.009	
	(0.031)		(0.017)	
Risk Score		0.013***		0.003
		(0.004)		(0.002)
Constant	0.134***	0.057**	0.028**	0.017
	(0.024)	(0.024)	(0.013)	(0.013)
Observations	1,443	1,469	1,443	1,469
R-squared	0.001	0.009	0.003	0.002

Notes: Robust standard errors in parentheses. *** p<0.01, ** p<0.05, * p<0.1.

2. Plans for professional screening and reasons for no professional screening

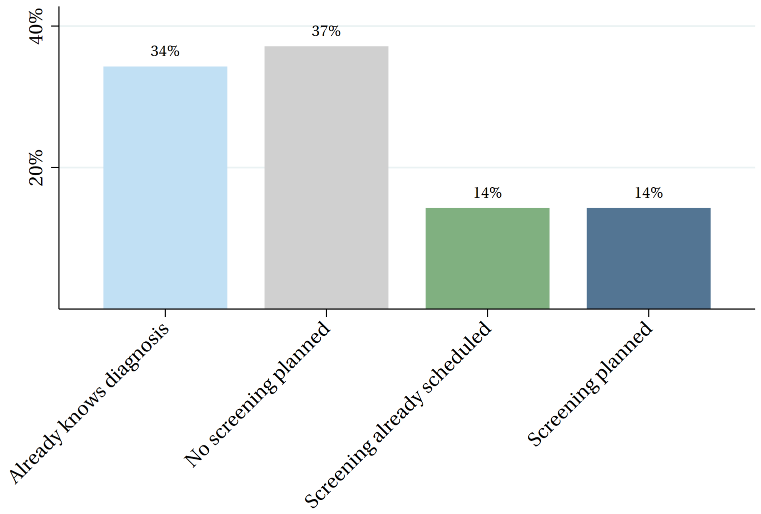

Supplementary Figure 1: Plans for professional diabetes screenings - The Figure shows the responses of high-risk individuals to the question whether they plan to comply with the received recommendation of scheduling a professional diabetes screening.

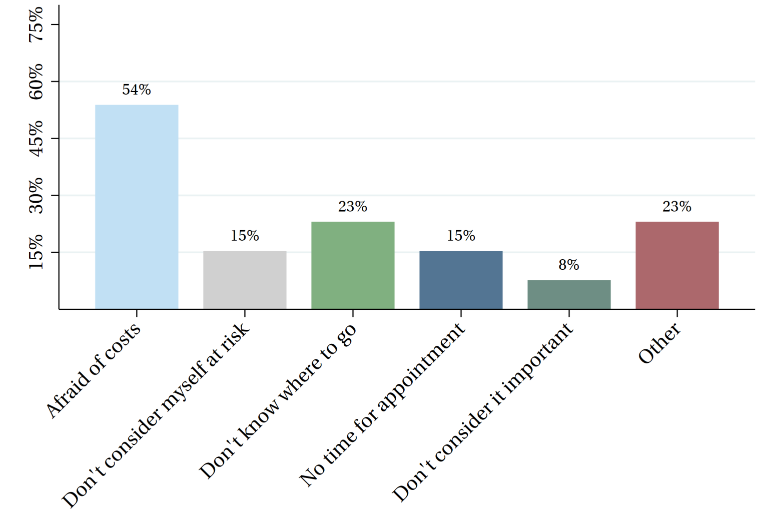

Supplementary Figure 2: Reasons for not planning a professional diabetes screening - The Figure shows the responses of individuals who answered they do not intend to schedule of professional screening despite having a high risk.

3. Details and results of the randomized framing

To account for a potential desirability bias in our survey, i.e., individuals may only report complying with the received recommendation because they expect this to be the socially desirable answer, we randomized two different framings of the same question.

Framing 1:
After completion of the test you received a recommendation to meet with a physician or GP to request a professional blood test for diabetes. Since it has already been 6 weeks since the completion of the risk test and given the urgency of a high diabetes risk, we assume that you have already scheduled an appointment with a doctor for a professional diabetes screening.

- Indeed, I have already scheduled an appointment.
- No, but I plan to schedule an appointment.
- I don't plan to schedule an appointment.
- I don't need an appointment since I already know that I have diabetes.

Framing 2:
After completion of the test you received a recommendation to meet with a physician or GP to request a professional blood test for diabetes. Since you completed the online diabetes risk test only 6 weeks ago, we assume that you may not have had sufficient time to schedule an appointment with a doctor for a professional diabetes screening.

- Indeed, but I plan to schedule an appointment
- I don't plan to schedule an appointment.
- I have already scheduled an appointment
- I don't need an appointment since I already know that I have diabetes.

Supplementary Table 8 reports the results of a chi-squared test to assess whether the response pattern to the question about the professional blood test differs between the two framings presented above. The results suggest that this is not the case, since the null-hypothesis of no significant correlation cannot be rejected (p-value 0.494).

Supplementary Table 8: Framing experiment

		Plans for scheduling professional blood test<br>Already knows diagnoses	No plans to schedule	Scheduled after screening	Plans to schedule	Total
**Framing**	Framing 1	4	8	3	3	18
	Framing 2	8	5	2	2	17
	Total	12	13	5	5	35

Pearson $\text{chi}^2(3) = 2.399$ Pr = 0.494

Supplementary Material 4: Additional tables and figures - ad heterogeneity

Supplementary Table 9: Results – Ad effectiveness (Regression coefficients - Logit model)

	(1) Link clicks	(2) Link clicks	(3) Link clicks women	(4) Link clicks men	(5) Conversion	(6) Conversion	(7) Conversion women	(8) Conversion men
**Reference: Family**								
Consequences	0.169***	0.221***	0.348***	0.107*	0.430***	0.462***	0.527***	0.391***
	(0.042)	(0.042)	(0.061)	(0.058)	(0.083)	(0.083)	(0.121)	(0.115)
Geography	-0.058	-0.019	0.075	-0.113*	0.249***	0.284***	0.380***	0.186
	(0.046)	(0.046)	(0.066)	(0.065)	(0.089)	(0.089)	(0.126)	(0.127)
Religion	0.114**	0.111**	0.172**	0.050	0.073	0.061	0.074	0.048
	(0.048)	(0.048)	(0.068)	(0.068)	(0.101)	(0.101)	(0.143)	(0.142)
Shock	0.134***	0.147***	0.219***	0.074	0.360***	0.366***	0.472***	0.253**
	(0.045)	(0.045)	(0.065)	(0.063)	(0.089)	(0.089)	(0.125)	(0.127)
Female		0.221***				0.218***		
		(0.028)				(0.053)		
**Reference: Below 45**								
45-54		0.583***	0.576***	0.585***		0.706***	0.750***	0.661***
		(0.034)	(0.047)	(0.048)		(0.062)	(0.087)	(0.087)
55-64		0.931***	0.883***	0.970***		0.752***	0.731***	0.766***
		(0.041)	(0.060)	(0.057)		(0.081)	(0.118)	(0.112)
65+		0.913***	0.862***	0.949***		0.209*	-0.026	0.334**
		(0.050)	(0.077)	(0.066)		(0.123)	(0.212)	(0.152)
Region (Yogyakarta =1)		-0.007	-0.065	0.044		-0.297***	-0.283***	-0.311***
		(0.028)	(0.041)	(0.039)		(0.055)	(0.079)	(0.076)
Observations	286,776	286,776	126,216	160,560	286,776	286,776	126,216	160,560
P-values of pairwise Wald test								
Consequences = Geography	0.000	0.000	0.000	0.000	0.016	0.019	0.179	0.049
Consequences = Religion	0.211	0.012	0.005	0.353	0.000	0.000	0.000	0.005
Consequences = Shock	0.383	0.069	0.031	0.562	0.348	0.201	0.615	0.188
Religion = Geography	0.000	0.006	0.148	0.017	0.061	0.018	0.021	0.302
Shock = Religion	0.681	0.444	0.481	0.712	0.002	0.001	0.003	0.124
Shock = Geography	0.000	0.000	0.025	0.003	0.174	0.318	0.418	0.564

Notes: Robust standard errors in parentheses. *** p<0.01, ** p<0.05, * p<0.1. The number of completed survey questionnaires counted as "conversion" are derived from the reduced sample without duplicated questionnaires (N=1,469) of which 1,443 could be linked to the referring ad theme. The missing 26 could not be linked to the referring ad theme due to tracking restrictions.

Supplementary Table 10: Results – Ad effectiveness (Marginal effects - Logit model)

	(1) Link clicks	(2) Link clicks	(3) Link clicks women	(4) Link clicks men	(5) Conversion	(6) Conversion	(7) Conversion women	(8) Conversion men
**Reference: Family**								
Consequences	0.0031***	0.0040***	0.0069***	0.0018*	0.0021***	0.0022***	0.0028***	0.0018***
	(0.0008)	(0.0008)	(0.0012)	(0.0010)	(0.0004)	(0.0004)	(0.0006)	(0.0005)
Geography	-0.0009	-0.0003	0.0013	-0.0017*	0.0011***	0.0013***	0.0019***	0.0008
	(0.0008)	(0.0007)	(0.0011)	(0.0010)	(0.0004)	(0.0004)	(0.0006)	(0.0005)
Religion	0.0020**	0.0019**	0.0031**	0.0008	0.0003	0.0002	0.0003	0.0002
	(0.0009)	(0.0008)	(0.0013)	(0.0011)	(0.0004)	(0.0004)	(0.0006)	(0.0005)
Shock	0.0024***	0.0026***	0.0041***	0.0012	0.0017***	0.0017***	0.0024***	0.0011**
	(0.0008)	(0.0008)	(0.0012)	(0.0011)	(0.0004)	(0.0004)	(0.0006)	(0.0005)
Female		0.0040***				0.0011***		
		(0.0005)				(0.0003)		
**Reference: Below 45**								
45-54		0.0093***	0.0103***	0.0084***		0.0034***	0.0041***	0.0029***
		(0.0006)	(0.0009)	(0.0007)		(0.0003)	(0.0005)	(0.0004)
55-64		0.0179***	0.0187***	0.0172***		0.0037***	0.0039***	0.0035***
		(0.0010)	(0.0016)	(0.0013)		(0.0005)	(0.0008)	(0.0006)
65+		0.0174***	0.0180***	0.0166***		0.0008	-0.0001	0.0012**
		(0.0013)	(0.0022)	(0.0015)		(0.0005)	(0.0008)	(0.0006)
Mean of dependent var.*	0.017	0.017	0.017	0.017	0.0039	0.0039	0.0041	0.0037
Observations	286,776	286,776	126,216	160,560	286,776	286,776	126,216	160,560

Notes: The Table shows the marginal effects of the logit models showsn in equation (1) and (2) in the main manuscript. Columns (2) and (6) correspond to the relative effects presented in Figures 2 and 3 in the main manuscript. Robust standard errors in parentheses. *** p<0.01, ** p<0.05, * p<0.1. The number of completed survey questionnaires counted as "conversion" are derived from the reduced sample without duplicated questionnaires (N=1,469) of which 1,443 could be linked to the referring ad theme. The missing 26 could not be linked to the referring ad theme due to tracking restrictions. *Mean of dependent var. refers to the mean of the dependent variable in the reference group.

Supplementary Table 11: Probability of attrition (OLS model)

	(1)<br>Attrited	(2)<br>Attrited	(3)<br>Attrited	(4)<br>Attrited
**Reference: below 45**				
45-54	0.084***		0.034*	
	(0.021)		(0.019)	
55-64	0.175***		0.108***	
	(0.029)		(0.028)	
65+	0.342***		0.231***	
	(0.042)		(0.045)	
Gender (Female=1)		-0.006	0.005	
		(0.018)	(0.018)	
**Reference: below 45#male**				
45-54#male				0.023
				(0.027)
55-64#male				0.096**
				(0.038)
65+ #male				0.243***
				(0.055)
Below 45#female				-0.007
				(0.028)
45-54#female				0.039
				(0.027)
55-64#female				0.114***
				(0.041)
65+ #female				0.190**
Constant	0.155***	0.185***	0.129***	0.135***
	(0.015)	(0.012)	(0.017)	(0.020)
Observations	2,052	1,873	1,873	1,873
R-squared	0.046	0.000	0.025	0.025

Notes: Supplementary Table 11 presents the probability of attrition conditional on having started the screening questionnaire. Robust standard errors in parentheses. *** p<0.01, ** p<0.05, * p<0.1.

Supplementary Material 5: Comparison of the sample and benchmark populations

In this section we describe to what extent our generated sample of participants who completed the screening questionnaire differs from the universe of people that met our eligibility criteria in Jakarta and Yogyakarta. To do so, we rely on different data sources: we use the official population statistics from the Indonesian Statistics Office (BPS)[6], we derive health data for prevalence rates of high blood sugar levels and high blood pressure as well as data on risk factors (smoking, physical activity, diet) for the population above the age of 35 from RISKESDAS 2018[7] and, lastly, we rely on primary survey data from the SUNI-SEA project – a large-scale, international policy and research project that implements NCD interventions in Southeast Asia and in which two of the authors are involved (MF and MG). From this project, we use data collected in the two urban regions of Kota Surakarta (Central Java) and Kota Kediri (East Java) on the topic of NCD screening. Although this sample does not constitute a representative sample for the cities Jakarta and Yogyakarta, it covers a randomly selected sample of individuals living in two urban areas on the same island as Jakarta and Yogyakarta (Java) and therefore provides an urban-Java-specific benchmark for the information that is not available in the representative sample of the RISKESDAS data.

Supplementary Table 12 compares the data collected in the screening questionnaire with the population benchmark data from BPS, RISKESDAS, or SUNI-SEA, depending on data availability. Column (1) contains the mean of the given variable from our generated data from the screening questionnaire, Column (2) presents the mean of the given variable from the comparison data, Columns (3) and (4) contain the information about the sample size and data source of the comparison data.

In comparison to the age distribution derived from the BPS statistics, the distribution generated in our experiment is slightly skewed toward the age group 45-55; especially the group above the age of 65 is underrepresented in our sample. This is, however, not surprising, given that Facebook is over-proportionally used by younger cohorts in Indonesia.[8] It means that a Facebook campaign such as ours is less suited to reach older generations as they are less often on social media platforms. In contrast, it can very effectively encourage the middle-aged, spanning 45-54 years of age, to conduct an online self-screening. This is an important finding, given that the risk of developing type 2 diabetes rises significantly after the age of 45 and regular diabetes screenings are recommended from the age of 45 onward by the Indonesian Endocrinology Association.[9] In terms of composition by sex, our sample can be considered statistically similar to the total population in Jakarta and Yogyakarta. There are no significant differences in the gender distribution between our data and the benchmark population.

Fifty percent of the respondents of the screening questionnaire claimed having ever been found to have had high blood sugar. There is no similar question in RISKEDAS for comparison. However, the share of individuals above the age of 35 having ever been diagnosed with diabetes by a doctor is equal to 3.5% in the total Indonesian population and 6% in Jakarta and Yogyakarta; the shares of individuals with diabetes and pre-diabetes according to blood testing in total Indonesia are 14% and 30%, respectively, implying that only 25% of the individuals with diabetes are aware of it. The sample from the SUNI-SEA project indicates that 62% have ever been screened for high blood sugar levels. Of course, we cannot make a statement about how the term “high blood sugar level” was interpreted by the respondents to our questionnaire, and having high blood sugar levels does not always imply (pre-)diabetes. However, such a high share indicating ever to have been found to have had high blood sugar implies a certain level of awareness about the possibility of having high blood sugar and a comparably higher risk of having diabetes in contrast to the benchmark population. Hence, taken

together, it seems that we over-proportionally attracted individuals who had at least once been tested for high blood sugar levels and had a positive test outcome. Similarly, the share of individuals having ever been diagnosed with high blood pressure or taking anti-hypertensive medication in our sample is significantly higher than in the benchmark population (33% vs. 16%). The same holds true for the risk factors obesity, daily physical activity, and daily fruit consumption. On average, the individuals in our sample are more likely to be obese, less likely to carry out sufficient physical activity, and less likely to consume fruits or vegetables on a daily basis compared to the benchmark population. Only the prevalence of current smokers is significantly lower in our sample than in the overall population.

Supplementary Table 12: Comparison of the sample with benchmark populations

	(1) Mean (sample)	(2) Mean (benchmark)	(3) Sample size (benchmark)	(4) Data source
Demographics				
Age			6,503,389	BPS 2022
35-45[1]	0.32***	0.34		
45-54	0.47***	0.29		
55-64	0.16***	0.21		
Above 65	0.05***	0.16		
Female	0.49	0.50	6,503,389	BPS 2022
Blood glucose				
Ever been found to have high blood sugar levels	0.50	-		
Ever diagnosed with diabetes (Jak. & Yog.)	-	0.06	11,599	RISKESDAS 2018
Ever diagnosed with diabetes (Indonesia)	-	0.035	418,187	RISKESDAS 2018
Diabetes according to blood test (Indonesia)[2]	-	0.14	25,767	RISKESDAS 2018
Pre-diabetes according to blood test (Indonesia)[2]	-	0.30	18,876	RISKESDAS 2018
Ever had blood sugar measured by health worker	-	0.62	775	SUNI-SEA 2021
Blood pressure / hypertension				
Ever diagnosed with high blood pressure	0.33***	0.16	10,062	RISKESDAS 2018
Hypertension according to measurement	-	0.34	10,494	RISKESDAS 2018
BMI				
Weight	69.25***	64.31	775	SUNI-SEA 2021
Height	162.36***	158.61	775	SUNI-SEA 2021
BMI	26.14**	25.61	775	SUNI-SEA 2021
BMI categories[3]			11,215	RISKESDAS 2018
Underweight (BMI <18.5)[1]	0.03***	0.07		
Normal (BMI ≥18.5 - <25.0)	0.44***	0.47		
Overweight (BMI ≥ 25.0 - <27)	0.18***	0.16		
Obesity (BMI ≥ 27)	0.35***	0.29		
Risk factors				
Daily physical activity	0.60***	0.68	11,598	RISKESDAS 2018
Smoking			11,778	RISKESDAS 2018
Never smoked[1]	0.66***	0.57		
Stopped smoking	0.20***	0.15		
Currently smoking	0.14***	0.28		
Daily fruit consumption	0.45***	0.93	11,791	RISKESDAS 2018

Notes: [1]Statistical differences in the distribution of categorical variables (age groups, BMI catgeories, smoking status) are assessed via Pearson's chi-squared test and significance stars are added to each category. [2]The data for the diabetes and pre-diabetes prevalence rates come from the full Indonesian sample above the age of 35, since data per province and age category was not available. [3]The BMI classifications are those provided in RISKESDAS. They differ from the scale used in the screening questionnaire, which relies on the risk-scale for BMI cut-off points for Asian populations provided by the World Health Organization Expert Consulation[10], in which overweight is classified as having a BMI above 23 instead of 25 and obesity as having a BMI above 27.5. *** p<0.01, ** p<0.05, * p<0.1.

Supplementary Material 6: Cost-effectiveness

In this section, we analyze the cost-effectiveness of our Facebook health campaign under the assumption that it would be scaled-up to a one-year health campaign across the whole island of Java. This implies a target population of about 25 million Facebook users above the age of 35. We perform a simple cost-effectiveness calculation based on the cost and effectiveness parameters derived from our study and enrich them with a repeated decision-tree model. The final cost parameter of interest is the cost per newly diagnosed person.

Supplementary Table 13, Panel A presents the cost indicators from our Facebook campaign averaged over the sub-groups: ad × gender × age group × region. Panel B presents the cost indicators by ad. The most relevant indicator is the cost per completed screening questionnaire, which is on average US$0.74, though with a large variation. The cheapest cost per completed questionnaire was achieved by the "consequences" ad, targeting females aged 45-55 living in Jakarta (US$ 0.24). Conversely, it was extremely costly to generate conversions, i.e., completed questionnaires, with the "religion" ad targeting males aged 65+ living in Yogyakarta (US$ 3.91). For a further analysis in terms of the campaign cost, we assume that the "consequences" ad is used exclusively, since it proved to be the most effective in terms of generating conversions (0.6% of Facebook users seeing the ad completed the screening questionnaire) and hence had on average the lowest cost per conversion (US$0.49).

Supplementary Table 13: Cost per view, reach, click and conversion (in US$)

	(1) Mean	(2) SD	(3) Min	(4) Max
**Panel A**				
Cost per view	0.001	0.000	0.001	0.003
Cost per reach	0.004	0.001	0.002	0.008
Cost per click	0.203	0.056	0.084	0.035
Cost per conversion	0.738	0.385	0.237	3.909

	(1) Cost per view	(2) Cost per reach	(3) Cost per click	(3) Cost per conversion
**Panel B**				
Ad design				
Family	0.001	0.004	0.226	0.987
Consequences	0.001	0.003	0.145	0.488
Geography	0.001	0.004	0.221	0.705
Religion	0.002	0.005	0.255	1.152
Shock	0.001	0.004	0.202	0.700

Notes: Supplementary Table 13, Panel A presents the summary statistics of the cost indicators, averaged over the sub-groups ad design × gender × age group × region. Panel B presents the cost indicators by ad design.

We use the average cost of US$2.84 per professional screening at an Indonesian primary health care facility drawn from Rattanavipapong *et al.*[11] Further, we rely on one assumption regarding the test sensitivity and two assumptions derived from the results of the screening questionnaire and our follow-up survey.

First, we assume that the screening questionnaire is adequately sensitive to detect diabetes, and

hence that the prevalence rate of diabetes in the "medium risk" and "low risk" group is 0%, whereas the prevalence rate in the "high risk" group is 80%. This sensitivity measure is well in line with that identified by Harbuwono *et al.*[12] for the American Diabetes Association risk test in Indonesia and by Rokhman *et al.*[5] for the Indonesian FINDRISC, who estimate a sensitivity of between 63% and 93%, depending on the cut-off level.

Second, we account for some form of self-selection, since we attracted a dis-proportionally high share of individuals that had ever been told they had high blood sugar levels (50% of participants) and a significant share that already had a diabetes diagnosis. Hence, to reflect the 34% of individuals with a high risk who indicated they did not need to conduct a professional follow-up screening since they already knew they had diabetes, we assume that 50% of the 80% of individuals that have diabetes (given the first assumption) are already aware of their diagnosis. This leads to a total of 40% of those individuals with a high risk already being diagnosed; slightly higher than the 37% identified in our study. This self-selection for campaign participation also implies that the total prevalence rate of diabetes in the campaign-participating population is slightly higher than in the non-participating population. Since the weighted prevalence rate for both groups (participating and not-participating) must be equal to the overall prevalence rate of 14% (reflecting the overall diabetes prevalence rate in Indonesia for the population aged 35 and above as identified in the RISKESDAS data), the prevalence rate of diabetes in the non-participating group is reduced to 13.78% and the ratio of diagnosed to undiagnosed cases is slightly lower (24.4%) than in the overall population.

Third, reflecting on the results of the follow-up survey, we assume that individuals with a "high risk" who are unaware of their disease status follow up with a professional diabetes screening in 40% of the cases (slightly less than the 43% found in our study).

Integrating the former assumptions, the cost and effectiveness measures and the risk distribution identified in our study, alongside the prevalence rates of diagnosed and non-diagnosed diabetes according to the RISKESDAS survey, leads to the final decision-tree depicted in Supplementary Figure 3.

We model the decision tree-flow for the total population of 25 million Javanese Facebook users above the age of 35 and as a monthly repeating intervention over the course of one year, i.e., all individuals who do not participate in the online screening questionnaire in the first (second, third etc.) month enter the decision-tree again in the second (third, fourth etc.) month. At the end of the decision-tree, an individual can have one of the following status: i) healthy, ii) diagnosed diabetes, or iii) undiagnosed diabetes. To be precise, the important difference between the results of the screening vs. the non-screening scenario is the distribution of individuals that have diabetes (14% of the total population) between the two states "diagnosed diabetes" and "undiagnosed diabetes".

The results of this repeated decision tree simulation are presented in Supplementary Table 14. The first row presents the results associated with the parameter assumptions outlined above ((i) the sensitivity of the online screening questionnaire, (ii) the share of individuals in the high risk group who are aware of their diabetes status, and (iii) the share of individuals with a high risk and unaware of their diagnosis who follow up with professional screening). We conduct multiple sensitivity checks by modifying the three above assumptions, first one by one and then all together, to see how they impact the final cost-effectiveness. The modified parameter is marked in bold in each row.

The main analysis reveals that the hypothetical up-scaling of the campaign to the whole of Java over the period of one year could lead to about 1.7 million users participating in the online screening, of whom about 250,000 would continue with the professional follow-up screening, and finally to the

diagnosis of almost 170,000 previously undetected diabetes cases. This corresponds to an increase from 25% to 29% of diagnosed cases relative to all cases, i.e., an increase of 16%. While the share might still seem small, the absolute number is large, especially in light of the low cost and low effort needed to implement an online health campaign. This low cost is further confirmed when we look at the total cost of the proposed intervention (including the professional follow-up screening), which is slightly higher than US$1.5 million. Dividing the total cost by the 170,000 newly diagnosed cases, the cost of detecting one more previously undiagnosed person amounts to approximately US$9.

Modifying several of the input parameters shows that the cost per diagnosed person barely surpasses a threshold of US$15. Even in the most pessimistic scenario (last row), with an assumed test-sensitivity of only 60%, with 75% of individuals receiving a high-risk score and having diabetes already knowing they have the disease, and only 20% of individuals following up with the recommendation, the cost for one newly diagnosed person amounts to only US$37.

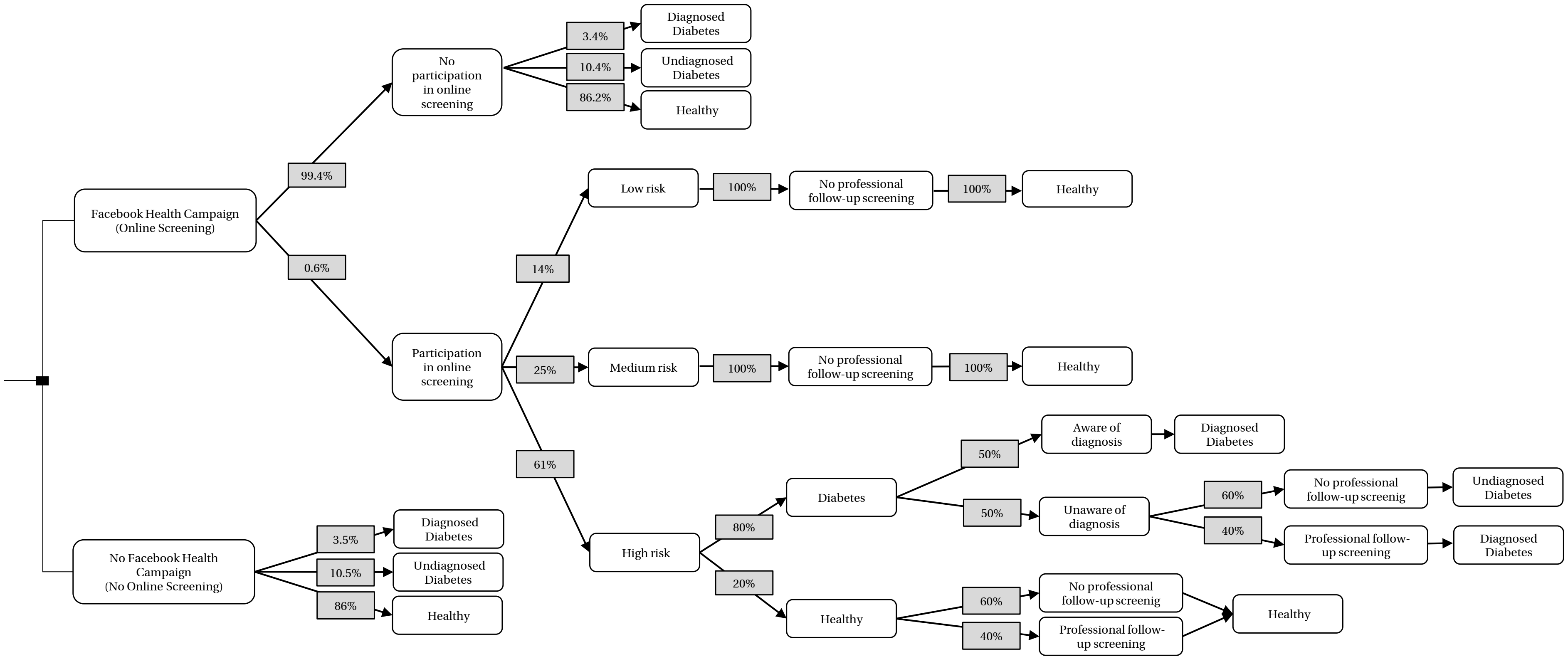

Supplementary Figure 3: Decision tree for states of diabetes diagnosis - The decision tree presents the different pathways and health states that individuals undergo in the “Online Screening Scenario” and the “No Online Screening Scenario”.

Supplementary Table 14: Cost-effectiveness results from the repeated decision tree with sensitivity analysis

	Assumptions			Costs		Results				
	(1)	(2)	(3)	(4)	(5)	(6)	(7)	(8)	(9)	(10)
	Sensitivity of online screening questionnaire	Share of individuals in high risk group being aware of diabetes status	Share of individuals with high risk following up with professional screening	Cost per online screening ($US)	Cost per professional follow-up screening ($US)	# of online screenings	# of professional follow-up screenings	Total cost ($US) (4)*(6)+(5)*(7)	Newly diagnosed cases	Cost per newly diagnosed patient ($US)
**Main analysis**	80%	50%	40%	0.49	2.84	1,741,772	254,995	1,574,171	169,997	9.26
**Sensitivity analysis**	**60%**	50%	40%	0.49	2.84	1,741,772	297,494	1,694,868	127,498	13.29
	**70%**	50%	40%	0.49	2.84	1,741,772	276,245	1,634,521	148,747	10.99
	**90%**	50%	40%	0.49	2.84	1,741,772	233,746	1,513,823	191,246	7.92
	80%	**25%**	40%	0.49	2.84	1,741,772	339,994	1,815,568	254,995	7.12
	80%	**75%**	40%	0.49	2.84	1,741,772	169,997	1,332,776	84,998	15.68
	80%	50%	**20%**	0.49	2.84	1,741,772	127,498	1,212,079	84,998	14.26
	80%	50%	**60%**	0.49	2.84	1,741,772	382,493	1,936,265	254,995	7.59
Best-case scenario	90%	25%	60%	0.49	2.84	1,741,772	494,053	2,253,095	430,305	5.24
Worst-case scenario	60%	75%	20%	0.49	2.84	1,741,772	116,872	1,181,901	31,874	37.08

Notes: Supplementary Table 14 shows the results from the repeated decision tree simulations. The first row presents the cost-effectiveness results when the input parameters are set as discussed in Section F. The following rows present the results when the input parameters are modified. The modified parameters are indicated in bold. In the last two rows, all three input parameters are modified to present a best-case and worst-case scenario.

Supplementary References

1. American Diabetes Association. American diabetes alert. *Diabetes Forecast* **46**, 54–55 (1993).

2. American Diabetes Association. Good to know: Diabetes risk test. *Clinical Diabetes* **37**, 291 (2019).

3. Lindstrom, J. and Tuomilehto, J. The diabetes risk score: A practical tool to predict type 2 diabetes risk. *Diabetes Care* **26**, 725–731 (2003).

4. Fauzi, N. F. M., Wafa, S. W., Ibrahim, A. M., Raj, N. B., and Nurulhuda, M. H. Translation and validation of American Diabetes Association diabetes risk test: The Malay version. *The Malaysian Journal of Medical Sciences* **29**, 113–125 (2022).

5. Rokhman, M. *et al.* Translation and performance of the Finnish Diabetes Risk Score for detecting undiagnosed diabetes and dysglycaemia in the Indonesian population. *PLOS ONE* **17**, e0269853 (2022).

6. Badan Pusat Statistik. *Population Indicators.* https://www.bps.go.id/en/statistics-table?subject=519 (2022).

7. Kementerian Kesehatan Republik Indonesia. *RISKESDAS 2018. Laporan Nasional Riskesdas.* https://repository.badankebijakan.kemkes.go.id/id/eprint/3514/1/Laporan%20Riskesdas%202018%20Nasional.pdf (2018).

8. Statista. *Share of Facebook users in Indonesia as of April 2021, by age group.* https://www.statista.com/statistics/1235773/indonesia-share-of-facebook-users-by-age/ (2022).

9. Soelistijo, S. *et al. Pedoman pengelolaan dan pencegahan diabetes melitus tipe 2 di Indonesia 2021.* Perkumpulan Endokrinologi Indonesia. https://pbperkeni.or.id/wp-content/uploads/2021/11/22-10-21-Website-Pedoman-Pengelolaan-dan-Pencegahan-DMT2-Ebook.pdf (2021).

10. World Health Organization Expert Consultation. Appropriate body-mass index for Asian populations and its implications for policy and intervention strategies. *The Lancet* **363**, 157– 163 (2004).

11. Rattanavipapong, W. *et al.* One step back, two steps forward: An economic evaluation of the PEN program in Indonesia. *Health Systems & Reform* **2**, 84–98 (2016).

12. Harbuwono, D. S., Mokoagow, M. I., Magfira, N., and Helda, H. ADA diabetes risk test adaptation in Indonesian adult populations: Can it replace random blood glucose screening test? *Journal of Primary Care & Community Health* **12**, 21501327211021015 (2021).
